# Neurological Complications of Local Anesthesia in Dentistry: A Review

**DOI:** 10.7759/cureus.50790

**Published:** 2023-12-19

**Authors:** Hashsaam Ghafoor, Saad Haroon, Sundus Atique, Anwar Ul Huda, Osman Ahmed, Ali O. Mohamed Bel Khair, Aijaz Abdus Samad

**Affiliations:** 1 Department of Anesthesia, Hamad Medical Corporation, Al Khor, QAT; 2 Department of Anesthesia, Qatar University, Doha, QAT; 3 Department of Endodontics, Primary Health Care Corporation, Doha, QAT; 4 College of Dental Medicine, QU Health, Qatar University, Doha, QAT; 5 Department Of Anesthesia, Hamad Medical Corporation, Doha, QAT; 6 College of Medicine, Qatar University, Doha, QAT; 7 Department of Anesthesia, Al Khor Hospital, Hamad Medical Corporation, Al Khor, QAT; 8 Department of Anesthesia and ICU, Latifa Women and Children Hospital, Dubai, ARE

**Keywords:** prevention, diagnosis, local anesthesia, regional nerve blocks, neurological complication

## Abstract

Local anesthesia is a technique that temporarily desensitizes a specific body area, typically for a surgical procedure, dental work, or pain management. It is described as a sensation loss in a specific area of the body due to depression of excitation in the nerve endings or due to the inhibition of the conduction process within the peripheral nerves. It allows for safer and more comfortable medical procedures, reducing the need for general anesthesia and facilitating faster recovery. Local anesthesia is generally safe, but like any medical intervention, it carries potential risks and side effects. The complications related to local anesthetics can be assessed in terms of neurological, vascular, local, systemic, and neurological. In this review article, we discussed the neurological complications of local anesthesia related to the ophthalmic nerve, maxillary nerve, mandibular nerve, branches of the trigeminal nerve, and facial nerve. These include diplopia, ptosis, paralysis of the eye, blindness, paresthesia, trismus, soft tissue lesions, edema, hematoma, facial blanching, infection, allergy, overdose, neuralgia, facial palsy, etc.

## Introduction and background

Neurological complications associated with local anesthesia (LA) in dentistry are rare but significant occurrences that warrant attention and consideration [1]. LA is commonly administered in dental procedures to ensure patient comfort and pain control during various dental treatments, such as root canal therapy, dental extractions, and pulp capping procedures. While the use of LA is generally considered safe, there have been reported cases of neurological complications that range from mild and transient to severe and long-lasting. Although these complications are uncommon, understanding and addressing them are crucial for providing safe and effective dental care [1].

## Review

Definition

LA is described as a reversible sensation loss in a specific area of the body due to depression of excitation in the nerve endings or the inhibition of the conduction process within the peripheral nerves [2].

Components

LA is composed of water-soluble salts derived from lipid-soluble alkaloids. They possess a structure comprised of three components, namely, lipophilic aromatic group, intermediary link, and hydrophilic amine group. LA is classified into amides or esters based on the intermediary link. By extending the length of the carbon chains connected to the amide linkage, tertiary amine, or aromatic ring, LA, such as lidocaine and bupivacaine, exhibits enhanced lipid solubility, potency, as well as extended duration of action (e.g., eutectic mixture of local anesthetics (EMLA) is a eutectic combination of 2.5% lidocaine and 2.5% prilocaine) [3].

Clinical uses

The use of LA is applied to various clinical scenarios in hospital practice [4].

Dental Procedures

LA is extensively used in dentistry to numb the teeth, gingival tissue, and surrounding tissues during various procedures. It is commonly administered by injection into the oral mucosa. Dental procedures such as tooth extraction, root canal treatment, dental fillings, and gingival treatments, including root planing and gingival grafts, often require the use of LA to ensure patient comfort [5].

Minor Surgical Procedures

LA is frequently employed in minor surgical procedures that involve small incisions or manipulations of tissues. Examples include skin biopsy, mole removal, wound repair, abscess drainage, and cyst excision. By blocking the sensation of pain in a specific area, LA allows for precise surgical interventions without the need for general anesthesia [6].

Obstetrics and Gynecology

LA is used in certain obstetric and gynecological procedures. It can be administered topically, as in the case of local anesthetic creams or gels used for pain relief during perineal repair after childbirth. Additionally, LA may be employed during procedures such as colposcopy, cervical biopsies, and minor vaginal surgeries [7].

Ophthalmology

LA is commonly used in ophthalmology to provide pain control during eye surgeries or interventions. Procedures such as cataract surgery, glaucoma surgery, and corneal transplant often involve the use of LA techniques, such as topical anesthesia with eye drops or peribulbar or retrobulbar injections around the eye [8].

Regional Anesthesia

LA plays a crucial role in regional anesthesia techniques, such as numbing large areas or specific nerves to block pain transmission during surgical procedures or for postoperative pain management. Examples include peripheral nerve blocks (PNB), epidural anesthesia, and spinal anesthesia. Regional anesthesia offers the advantage of providing prolonged pain relief and reducing the need for systemic analgesics [9].

Dermatology

LA is utilized in dermatology for procedures such as skin excisions, cryotherapy, laser treatments, and filler injections. Numbing the area with LA ensures patient comfort during these dermatological procedures and allows for precise manipulation of the skin or subcutaneous tissues [10].

Pain Management

LA techniques can also be used for diagnostic and therapeutic purposes in pain management. Procedures such as trigger point injections, joint injections, and nerve blocks can provide targeted pain relief for chronic pain conditions, such as neuropathic pain, trigeminal neuralgia, myofascial pain syndrome, and arthritis [11].

Types of complications

Vascular

Vascular complications due to LA are relatively rare. These complications can occur if the solution of local anesthetics is accidentally injected into the blood vessel or if vasoconstrictors are used improperly. Injection into a blood vessel can lead to systemic toxicity or localized ischemia.

Local anesthetics are vasodilators, hence the addition of a vasoconstrictor like adrenaline provides the following advantages: improves the anesthetic onset and duration, reduces bleeding, and decreases the systemic absorption rate of local anesthetics by reducing the plasma concentration. However, when used inappropriately, vasoconstrictors can compromise blood flow to the surrounding tissues. Prompt recognition and appropriate management are crucial in minimizing the risks associated with vascular complications. Adherence to proper injection techniques, aspiration to rule out intravascular injection, and knowledge of anatomical landmarks help reduce vascular complications incidence during LA procedures [5,12,13].

Local

Frequent local complications related to LA are pain at the injection site, needle fracture, anesthesia prolongation and several sensory disorders, trismus, lack of effects, infectivity, soft tissue injury, gingival lesions, hematoma, edema, as well as ophthalmologic complications [14-16].

Systemic

Frequent systemic reactions caused by LA are described as psychogenic reactions, allergy, methemoglobinemia, and systemic toxicity [14-16].

General Complications

General complications comprise ocular, neurological, allergy, hematoma, osteomyelitis, ankylosis, needle breakage, blanching, tissue necrosis, isolated arterial fibrillation, pregnancy-related complications, etc.

Ocular

Numerous instances of visual disruptions have been reported after the use of dental LA [17]. Diplopia is described as an unfavorable outcome; however, it can be resolved after 15 minutes to 24 hours [18-20]. Partial or complete vision loss, strabismus, uniocular blindness, and blurred vision were reported as well, but all these were transient [21-24]. In their study, Rishiraj and colleagues [25] reported a case of permanent vision loss in one eye after administration of a combined technique involving infiltration anesthesia and inferior alveolar nerve block (IANB). Vision loss was reported by patients in the left eye after the removal of several teeth with the administration of prilocaine. This issue was not resolved even after two months following the procedure.

Several studies have reported eye muscle disturbance as well. Ngeow and coworkers [26] reported two cases in which patients experienced accommodation loss in the eyes on the same side following IANB. Two studies reported cases where patients exhibited either incapability to abduct their eyes or underwent limitations in eye abduction [19,27]. In a study, Penarrocha-Diago and associates [28] reported 10 patients who experienced palsy of the external rectus muscle and one patient who exhibited incapability to look downwards due to superior oblique muscle impairment. Posteriorly dislocated eyeballs due to eye muscle function loss have been reported as well.

In various studies, eyelids dropping has been documented [22,28,29]. Furthermore, at least one study reported incapacity in fully closing the eyelids [30]. In a study, Goldenberg [31] described the development of numbness in eyebrows and eyelids after infiltration anesthesia. In two studies, pupil excessive contraction was described while pupil excessive dilation has been reported as well [28,29,32]. The burning sensation and pain have been reported by Uckan and colleagues [23] after the administration of dental LA. The overall anticipated prevalence of ocular unfavorable outcomes was between 0.07% to 0.09% [28].

Neurological

Neurological complications associated with LA are rare but important to be aware of. These complications can include nerve injury, neurotoxicity, transient neurological symptoms (TNS), and central nervous system (CNS) effects. Nerve injury can occur due to trauma during needle insertion or local anesthetic toxicity. Neurotoxicity can manifest as TNS or persistent neurological deficits. CNS effects can occur if high systemic levels of local anesthetics are reached. Prompt recognition and appropriate management are essential in minimizing the risks associated with neurological complications [12,13].

Several studies have reported cases of trigeminal nerve injury due to the administration of dental LA, ranging from mild and transient to acute and permanent [17]. Hillerup and colleagues [33] reported 54 patients who had cranial nerve (CN) V injury following IANB administration. Among 77.8% and 22.2% of cases, lingual and inferior alveolar nerves (IANs) were affected, respectively. The nerve injury symptoms comprise allodynia, dysesthesia, and paresthesia. A study conducted by Garisto and teammates [34] documented lingual and IAN involvement in 89% and 11% of cases, respectively. In a study, Kingon and fellows [35] reported five cases of paresthesia and dysesthesia following mental nerve block or IANB using anesthetic solutions. A case of nerve permanent injury with hearing loss, facial numbness, ataxia, and facial palsy was reported on the same side of the injection [36]. The total prevalence of nerve permanent injury due to mandibular LA was between 0.000007% and 0.003% [34,37]. Following infiltration anesthesia, Moorthy and colleagues [38] reported a paresthesia case in the maxillary region. Patients felt numbness in the upper lip while the gingiva was in the anterior left area of the maxilla [38]. A singular case highlighting inflammatory trigeminal lesions was reported. The symptoms comprised numbness and paresthesia of the face, lip, tongue, forearm, and hand on the same side, but all were transient [39].

Allergy

After the dental LA, several allergic reaction cases have been described [17]. Allergenic responses can be triggered by additives such as metabisulfite or methylparaben, which can be found in dental LA [40]. The local anesthetics that are associated with allergic reactions comprise lidocaine, lidocaine, diphenhydramine hydrochloride, articaine, mepivacaine, prilocaine, and procaine [41-46]. A case of anaphylactic shock after administration of lidocaine was documented. After 20 minutes of LA administration, facial edema occurred, orbits were found closed, and urticaria was visible on the cheek. The patient encountered slight respiratory problems [47].

Allergy due to articaine was documented in two studies [42,48]. The symptoms comprised erythema, dizziness, facial edema, palpitations, chest pain, respiratory distress, discomfort, and redness & itching on the hands, chest, and abdomen. A case of allergenic reaction with diphenhydramine injection as local anesthetics was documented. Diffuse inflammation occurred on the injection side after 24 hours of administration [43]. Ross and collaborators [49] documented two cases of serum-type allergenic reactions with mepivacaine. The first patient had a headache, nausea, vomiting, malaise, and fever while the other had local edema, joint pain, nausea, and headache.

Hemorrhage/Hematoma

Bajkin and associates [50] carried out a study to evaluate safety of the dental LA among patients using anticoagulants. The study found only two minor hematomas while no protracted hemorrhage. Dougall and comrades [51] assessed the safety of the buccal infiltration anesthetic agent among patients suffering from hemophilia. After buccal infiltration administration, no hematoma above 2 mm was documented. Moreover, no differences were reported in the superficial hemorrhage times based on hemophilia severity or healthcare practitioner experience. Brodsky and their companions [52] documented a case of ear problems after the Gow-Gates injection. Increased pressure was experienced by patients in the ear region and hearing problems such as sudden sensorineural hearing loss (SSHL), which is an acute hearing loss. A study carried out by Srisurang and collaborators [53] compared the effectiveness and unfavorable outcomes of mepivacaine, articaine, and lidocaine and found no severe unfavorable outcomes; however, only two ecchymoses were noticed with articaine at the injection site one hour after LA administration.

Injury to an IAN can occur during a traumatic LA injection. Although very rare, hematoma formation can occur. The needle may traumatize the epineural blood vessels. Hemorrhage from the epineural blood vessels would compress the nerve fibers and cause localized neurotoxicity [17,18]. The damage could be extended beyond 30 minutes after injection. The release of blood and blood products from the epineural blood vessels into the epineurium during hematoma formation would lead to reactive fibrosis and scar formation, applying pressure to and inhibiting the natural healing of the nerve [17,18].

Needle Breakage

During administration, needle breakages of local anesthetics can take place [17]. Pogrel [54] reported needle fractures in 16 cases that were documented during a period of 25 years. However, this study included only four cases due to inclusion criteria (age >18 years). The needle fracture after an IAN block was reported in three cases. Pogrel [54] reported that the prevalence of needle fracture was 0.000007%. Zijderveld and teammates [55] documented a case of needle fracture following an IAN block. Rahman and colleagues [56] described a case of a 65-year-old person wherein a needle fracture occurred during IANB administration.

Osteomyelitis

Barnard [57] reported an osteomyelitis case after infiltration anesthesia within the maxilla. A week after administration, there was a development of fluctuating swelling in the cheek along with pain. Trismus was evident while the mouth opening was restricted to 8 mm. Additionally, it was confirmed that there was mental nerve paresthesia on the same side. Radiographs showed a "moth-eaten" appearance of ramus, condyle, and coronoid process on the same side [57].

Tissue Necrosis

Woodmansey and his research team [58] described an osteonecrosis case after intraosseous anesthesia. Satisfactory anesthesia with conservative IAN block was challenging, so the decision was taken to perform intraosseous anesthesia. During perforating cortical bone, the metal perforator got stuck at the injection site and was detached from the plastic base. After the incision, an insignificant part of the bone was eliminated for the retrieval of the metal perforator. The findings of another study demonstrated a skin necrosis case after dental LA. Three hours later, the patient experienced a sensation of stinging in his mouth. Skin erosion was observed rapidly, leading to the immediate scheduling of follow-up appointments. After three days, the signs completely vanished [59].

Blanching

Webber and partners [60] described a blanching case after the administration of the IAN block. The patient complained of light-headedness and dizziness shortly following the administration. Visible paleness was noticed in the infraorbital area, nose, lip, and lower eyelid, accompanied by hemifacial numbness experienced by the patient. A further case report indicated skin paleness above the lip at the injection site following the IAN block [61].

Ankylosis

Luchetti and associates [62] performed a study to investigate the association between dental injections of local anesthetic and jaw ankylosis among patients who had fibrodysplasia ossificans progressiva. After LA administration, five patients complained of permanent jaw ankylosis and ossification. The symptoms exhibited by the patient included stiffening and pronounced inflammation of the jaw, resulting in loss of movement [62].

Isolated Atrial Fibrillation

Manani and colleagues [63] reported an isolated atrial fibrillation case following dental LA administration. The patient was administered bilateral maxillary infiltration using mepivacaine and complained of symptoms such as light-headedness, abdominal discomfort, and palpitations. Electrocardiogram results revealed isolated atrial fibrillation, which resolved about 12 hours later [63].

Pregnancy-Related Adverse Effects

A comparative study carried out by Hagai and colleagues [64] evaluated the adverse effects associated with pregnancy after dental LA administration. Among two groups of patients, the related numbers of main abnormalities were compared. Intrauterine neoplasms, twin-to-twin transfusion conditions, and cardiac septal abnormalities were described as the main abnormalities. The frequencies of ectopic pregnancy, miscarriage, induced abortion, and stillbirth were compared as well. Exposure occurred in the experiment group during the first (53%) and 2nd trimesters (45%). The exposure was unknown in 2% of cases. The frequency of main abnormalities among experimental group patients was 4.8% versus 3.3% among control group patients with statistically no differences (P = 0.300) [64].

Neurological complications

The most common nerves involved during dental procedures under LA are the fifth cranial nerve (trigeminal nerve) and the seventh cranial nerve (facial nerve) can have potential implications for neurological complications when using LA.

Trigeminal Nerve

**Figure 1:**
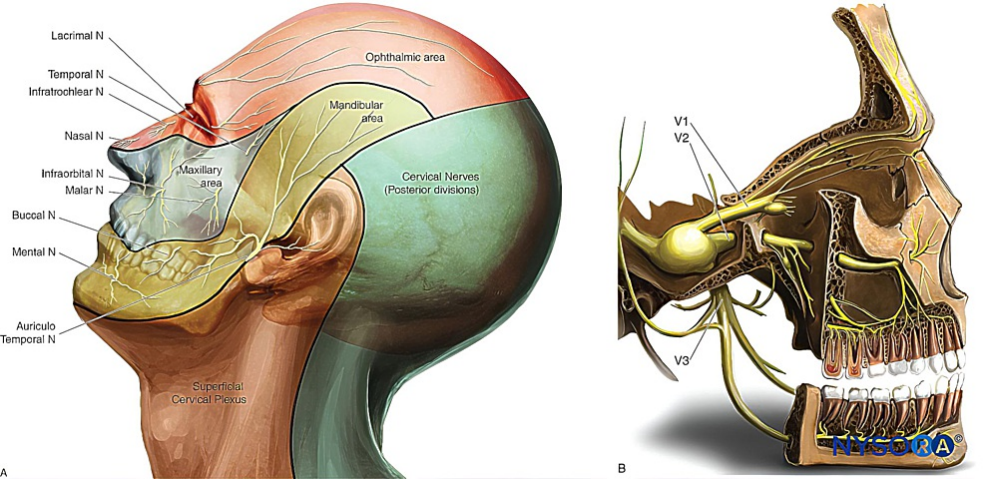
Innervation of the face. (A) Dermatomes of the head, neck, and face. (B) Distribution of the three branches of the trigeminal nerve. Used with permission from [65].

Sensory innervation of the face and neck is supplied by the trigeminal nerve (fifth cranial or V) [65]. It plays an important part as a primary pathway for sensory input from the face [66]. The fifth cranial nerve carries both sensory and motor components. The trigeminal ganglion (semilunar or Gasserian ganglion) lies in Meckel’s cave, an invagination of the dura mater near the apex of the petrous part of the temporal bone in the posterior cranial fossa. Postganglionic fibers exit the ganglion to form three nerves (Figure 1) [65].

Ophthalmic (CN V1)

The trigeminal nerve, the first division of the ophthalmic nerve, exits the trigeminal ganglion in lateral, forward, and upward directions to traverse the cavernous sinus [67]. Subsequently, the ophthalmic nerve enters the orbital cavity via a superior orbital fissure. It then further divides into three main branches, namely, frontal, nasociliary, and lacrimal nerves. Each of these three branches carries sensory fibers to distinct regions of the forehead, eyelids, nose, and scalp, respectively [65,67].

Maxillary (CN V2)

It is the second division of cranial nerve V that leaves trigeminal ganglion in horizontal, forward, and somewhat lateral directions [68]. The maxillary nerve runs via the cavernous sinus as well. It then descends and reaches the foramen rotundum before entering the pterygopalatine fossa where maxillary nerves divide into several branches, namely, infraorbital sphenopalatine, palatine, and zygomatic branch. Both zygomatic and infraorbital nerves provide sensory innervation to the midface area (cheek, nose, upper lip, and anterior temporal part) [65,68].

Mandibular (CN V3)

The mandibular nerve, the third division of the fifth cranial nerve, exits the trigeminal ganglion and descends downwards [69]. The mandibular nerve is an assorted sensory-motor nerve that possesses the smallest intracranial segment among the three divisions of the fifth cranial nerve. When leaving the middle cranial fossa, the mandibular nerve traverses the foramen ovale, entering the infratemporal fossa, where it promptly splits into two branches (anterior trunk and posterior trunk). Anterior trunk consists of motor branches that connect to the masticatory muscles, as well as the long buccal nerve responsible for providing sensory innervation to buccal mucosa and cheek. The posterior trunk, which is larger, encompasses the lingual, inferior alveolar, and auriculotemporal nerves. In the infratemporal fossa, chorda tympani (which comprises secretomotor fibers and special sensory/taste afferents) forms a neural link with the facial nerve, joining the latter. In the facial soft tissue, sensory innervation is provided by the auriculotemporal nerve to the auricle, tragus, and posterior portion of the temple. Sensory innervation is provided by the inferior alveolar to the chin and lip together with mental as well as branches of the mylohyoid nerve [65,69,70].

Facial Nerve

The 7th cranial nerve emerges from the skull via the stylomastoid foramen. Before the emergence of facial nerve from the skull, the chorda tympani branch off at the petrous bone level. It supplies the preganglionic secretomotor fibers to sublingual and submandibular salivary glands. Also, it carries efferent taste fibers from the frontal two-thirds of the tongue, excluding lingual papillae. When it exists in the skull, the facial nerve bifurcates into two major branches, i.e., cervical and temporal, before entering the parotid gland, where it continues to split into temporal, buccal, zygomatic, cervical, and mandibular branches. These supply the mimic muscles [71].

Other Nerves

Lingual nerve: A specific branch that originates from the mandibular division of the trigeminal nerve (V3) is responsible for providing sensory innervation to the front 2/3 of the tongue [12,72].

Inferior alveolar nerve (IAN): It is a mandibular division branch of the trigeminal nerve (V3) that innervates the lower teeth, chin, and lower lip [12,72].

Mental nerve: A terminal branch of IAN that provides sensory innervation to the lower lip and chin [12,72].

Posterior superior alveolar nerve (PSAN): The maxillary division of the trigeminal nerve (V2) innervates the upper molars and premolars [12,72].

Signs of Neurological Complications

There are many signs of neurological complications like complete or partial lack of sensation within the affected area, prolonged numbness of the tongue, cheek, or gingival tissue, loss of sensation beyond the expected duration after LA administration, numbness of the jaw and face, tingling sensation in the affected area, and unusual or persistent pain or burning sensation in the area where the LA was administered [12,72].

Symptoms of Neurological Complications

There are many symptoms of neurological complications like agitation, restlessness, irritability, or a state of increased anxiety, confusion, difficulty in thinking, impaired judgment, disorientation, dizziness, a sensation of light-headedness, feeling faint, drowsiness, feeling excessively sleepy or fatigued, and a metallic taste in the mouth [5,73].

Incidence of Neurological Complications

Estimating the exact prevalence of neurotoxicity caused by local anesthetics is challenging because of the presence of several confounding risk factors that can cause nerve injury during the perioperative period [74]. Based upon several studies focusing on PNB, the neurological incidence of complications related to this technique was generally found to be below 3%. The majority of these complications are temporary sensory loss while persistent nerve injury is uncommon [75-77]. Several research studies on neurological problems with PNB have demonstrated that nerve injury risk was between 0.02% and 0.5%. The neurotoxicity incidences due to local anesthetics differ among research because the assessment regarding neurotoxicity incidence caused by local anesthetics is induced by techniques utilized for the measurement of neurological complications associated with anesthetics [76,77]. Urban and Urquhart [78] assessed that neurological deficit incidence was 3% to 5% following a survey carried out regarding neurological deficits two weeks after the brachial plexus block. However, the neurological deficits incidence beyond four weeks was just 0.4% [78]. An elevated risk of protracted paresthesia was observed after 4% articaine administration than after other anesthetic drug administration [33,79]. Hillerup and teammates [79] indicated that 4% of articaine leads to neurosensory problems in two trigeminal branches. Furthermore, neurosensory disturbances related to 4% of articaine were mainly associated with mandibular blocks [79].

Ophthalmic nerve

The ophthalmic nerve is a branch of the 5th cranial nerve and plays a crucial role in innervating the eye and surrounding structures. When LA affects the ophthalmic nerve or its branches, it can cause various effects on the eye and periorbital region. Some of these effects include direct effects on the eye and periorbital effects (Tables 1, 2) [5,80].

**Table 1:** Direct effects on the eye.

Diplopia	Local anesthesia affecting the ophthalmic nerve can disrupt the normal function of the extraocular muscles, leading to double vision or diplopia.
Ptosis	Ptosis, characterized by upper eyelid drooping, can occur due to the effects of local anesthesia on the muscles responsible for eyelid elevation.
Mydriasis	Local anesthesia can cause dilation of the pupil (mydriasis) by affecting the iris muscles and interrupting the parasympathetic innervation of the pupil [5,80].

**Table 2:** Periorbital effects.

Facial paralysis	Local anesthesia in the periorbital region can potentially affect the facial nerve (cranial nerve VII) and result in facial muscle weakness or paralysis.
Periorbital blanching	Local anesthesia can cause temporary vasoconstriction in the periorbital blood vessels, leading to a pale or blanched appearance around the eye [5,80].

These effects are typically transient and reversible once the local anesthetic wears off [5,80]. Several factors responsible for ocular complications caused by intraoral LA are diffusion, venous injection, unintentional needle penetration in the orbit, retrograde arterial injection, embolism, and sympathetic impulse generation [81-83].

The nerve blocks that are known to be related to ocular complications are the IAN block and PSAN block [81]. In the last 45 years, the ocular complications following middle/PSAN blocks have been reported to occur twice as frequently as those after IANBs. The PSAN is a maxillary division branch of cranial nerve V originating in the pterygopalatine fossa just before entering the infraorbital canal. It has two divisions that provide the sensory innervation to the maxillary sinus mucous membrane and the other supplies to periodontal ligaments, alveoli, and pulpal maxillary molars tissues. The PSAN block is mostly administered to attain anesthesia of the maxillary molars as well as adjoining structures [81].

The IAN is the major branch of the trigeminal nerve’s mandibular division. It moves down the lateral pterygoid muscle & anteroposterior to the lingual nerve and moves up the mental foramen wherein it provides its terminal divisions, i.e., mental and incisive nerves. Throughout its trajectory, the IAN is consistently accompanied by an inferior alveolar vein. This artery is positioned anteriorly to the nerve. Mostly the IAN block is given to attain anesthesia of the mandibula teeth as well as adjoining structures [81].

The symptoms that are commonly experienced are double vision, blurring of the vision, squinting, vision loss, reduced feeling on the lateral aspect of lower & upper eyelids, upper eyelid dropping, transient dizziness, and intricacy in reading caused by accommodation paralysis [81].

Complications of Ophthalmic Nerve

Diplopia: Ocular complications are mostly a result of LA accidentally administered intravascularly. Due to the proximity of the inferior alveolar artery to IAN, there is an elevated risk of intravascular injection. As a result of the high pressure applied during the injection of an anesthetic agent, it can forcefully be redirected toward the maxillary artery. The middle meningeal artery (MMA), which originates from the maxillary artery, is closely positioned to the point of the origin of the inferior alveolar artery. This proximity increases the probability of an anesthetic drug entering the MMA. The ophthalmic division of the MMA has the potential to establish an anastomotic connection with the lacrimal artery. The supply of blood to the lateral rectus muscle originates from both the lateral muscular trunk and lacrimal artery of the ophthalmic artery. It can thus cause paralysis of the lateral rectus leading to diplopia [84].

Inadvertent introduction of the anesthetic drug in the venous system, leading to drainage in the pterygoid plexus and subsequently the cavernous sinus can result in diplopia. The abducens nerve is very vulnerable because it traverses via the cavernous sinus causing lateral rectus muscle paralysis leading to diplopia [85].

Paralysis of the eye: Inadvertent introduction of the local anesthetic agent in the venous system can cause drainage in the pterygoid plexus and then in the cavernous sinus. The backflow in the cavernous sinus can create pressure within the sinus leading to oculomotor nerve paralysis as the oculomotor nerve enters the roof of the cavernous sinus slightly lateral and anterior to the dorsum sellae [86,87].

Blindness (amaurosis): The central retinal artery (CRA), a branch of the ophthalmic artery, can be affected if the anesthetic drug flows through MMA. It can potentially result in blindness and pupillary light reflex loss, as the drug may travel into the ophthalmic artery and then reach the retinal artery [81].

The CRA reflex vasospasm causes ischemia and retinal tissue necrosis leading to permanent blindness [88]. To date, there is limited documentation regarding permanent blindness caused by intraoral LA. De Keyzer and colleagues [89] and Rishiraj and fellows [25] demonstrated perpetual blindness in one eye after administration of the intraoral LA. In this case report, the authors revealed that the patient was diagnosed with bacterial endocarditis and suffered permanent visual loss from the left eye after undergoing dental extraction before their mitral valve surgery. It was suggested that the complication could be avoided If the delivery of LA had been conducted with aspiration before and during the injection thus allowing the prevention of intravascular injection and leading to fluid emboli that may have occluded the ophthalmic artery with the devastating result of vision loss.

Ptosis, strabismus, and loss of accommodation: The ability to accommodate is dependent upon the lens capsule elasticity as well as ciliary muscle contraction that are innervated through short ciliary nerves, which are ciliary ganglion postganglionic fibers and are provided by the parasympathetic preganglionic fibers through an oculomotor nerve. Once the oculomotor nerve is paralyzed due to anesthesia or injury, it leads to ptosis, pupil dilatation, strabismus, and accommodation loss as the ciliary muscle, internal rectus, and sphincter pupillae are paralyzed. Partial nerve paralysis is a possible occurrence in certain cases. Internal strabismus may arise due to spasms in the internal rectus muscle, accommodation could be limited to nearby objects just caused by ciliary muscle spasm and miosis may take place due to sphincter irritation of the pupil [81]. Literature indicates similarities with Horner's syndrome, characterized by ptosis, conjunctiva vascular dilatation, miosis, and a widespread rash covering the face, arm, neck, and shoulders. The proposed mechanism is the inadvertent injection of a local anesthetic agent in the stellate ganglion [90].

Maxillary nerve

It is one of the three major branches of the fifth cranial nerve and plays an important part in the transmission of sensory information from the face to the brain. It carries sensory fibers responsible for touch, pain, and temperature sensations from various structures in the face. The maxillary nerve receives sensory input from the trigeminal ganglion maxillary division, which is located within the skull. It exits the skull via the foramen rotundum and branches into smaller nerves that innervate specific areas. Touch sensations are carried by the maxillary nerve, allowing one to perceive light contact or pressure on the face. Pain sensations from structures such as the maxillary teeth, maxillary sinuses, nasal cavity, and palate are transmitted through this nerve. Also, the maxillary nerve carries temperature sensations and enables it to perceive hot and cold stimuli in the areas it innervates [91-93].

Complications of Maxillary Nerve Block

Paresthesia: Paresthesia can be a potential complication of a maxillary nerve block (MNB), although it is relatively rare. Paresthesia refers to abnormal sensations, for example, numbness, tingling, or a "pins & needles" feeling, that occur in a particular area of the body. In the case of the maxillary nerve, paresthesia can take place due to nerve trauma/presence of a local hematoma (accumulation of blood) [94]. Maxillary nerve trauma, which can result from facial trauma, surgical procedures, or other causes, can lead to nerve damage or compression. This can disrupt the normal transmission of sensory signals, causing paresthesia in the innervated areas of the face.

Similarly, the presence of a local hematoma, which is a collection of blood in a specific area, can exert pressure on the maxillary nerve, leading to paresthesia. Hematomas can occur as a complication of surgical procedures, facial fractures, or other forms of trauma. Recovery from paresthesia resulting from maxillary nerve trauma or a local hematoma can take time. It is generally reported that it may take between six and 12 months for the nerve to heal and for normal sensation to be restored. However, the exact recovery period can differ depending on the extent of the nerve injury, individual factors, and the underlying cause of the paresthesia.

Facial nerve paralysis: Facial nerve palsy (FNP) is infrequent but described as a potential complication of MNB anesthesia. The actual prevalence is not known [95]. In the literature, most cases with immediate palsy recovered within seven hours. Occasionally the onset may be delayed and can lead to an extended recovery period [95].

Immediate type, rapid recovery: The majority of the reported cases of FNP caused by IANB anesthesia are characterized as immediate onset, with a tendency for swift recovery. Some of the different probable primary mechanisms are direct trauma caused by the needle, formation of hematoma with subsequent nerve compression, air blast incidence during surgical treatment, and infiltration of facial nerve peripheral branches owing to local anesthetic agents [95-97].

Delayed type: The following are the suggested possible factors regarding delayed FNP following IANB anesthesia [95]: (a) recurrence of the latent viral infectivity, for example, herpes zoster and herpes simplex. Local trauma can potentially serve as a triggering factor for viral replication, subsequently causing inflammation and dysfunction of the neural sheath [98]. (b) Axonal ischemia can occur due to a late reflex spasm in the facial nerve’s vasa nervorum. The vasa nervorum are the source of nutrition supplying each peripheral nerve. They arise from the adjacent blood vessels. These nutrient vessels are tortuous, which allows them to have considerable freedom of translational movement within the peripheral nerves, particularly in the vicinity of joints. It is believed to be attributed to a sympathetic vascular reflex activated through stimulation of the sympathetic plexus located along the external carotid artery. During nerve block, needle mechanical action, anesthetic drug infiltration, and epinephrine or breakdown products of anesthetic drugs all have been proposed as contributory factors [14,99]. (c) Facial nerve excessive stretching resulting from extended oral instrumentation causes ischemia or direct damage [96]. (d) Anesthetic drug intra-arterial inadvertent injection. It has been demonstrated to cause retrograde flow throughout the arterial system, leading to anesthetic drug distal spread, including central regions, and subsequent complications because of this phenomenon [14].

Trismus: Trismus may be a complication of MNB. It is commonly referred to as lockjaw and can cause decreased opening due to muscle spasms. Numerous factors lead to trismus, for example, several injections in the same region within a short time, intramuscular injections in the muscle that lead to the formation of hematoma, needle breakage within muscles introducing to the styloid process, wrong needle positioning during administration of inferior nerve block and low-grade infectivity [15].

Soft tissue lesions: Soft tissue lesions due to MNB are uncommon. Tissues such as the tongue, lips, gums, and lips if injured accidentally due to being bitten while eating or trauma. Chewing on hard objects can also damage soft tissues and may lead to persistent, throbbing pain. Soft tissue lesions that may occur following an MNB can be due to several factors, including needle trauma, infection, and allergic reactions, including developing sudden rashes, itching, facial swelling, or difficulty breathing if there is an allergic reaction during regional anesthesia or sedation [15,100-103].

Edema: Edema, or swelling, directly resulting from an MNB is uncommon. Inflammation can be caused by trauma during injection, allergy, infectivity, hemorrhage, as well as irritating solutions injection. The edema caused by trauma should be treated like a hematoma. Among the cases of edema caused by infection, the recommended course of treatment involves antibiotics prescription [15,100-103].

Facial blanching: Facial blanching, or pallor of the skin, is not a common or expected complication of MNB; however, it has been reported as a complication of LA in dentistry. Affecting the terminal branches of the maxillary artery, being a reversible end-organ phenomenon, explained by the theory of sympathetic vasospasm, and being observed in the distribution of the infraorbital artery are the specific principles of facial blanching [104,105].

Hematoma: Hematoma, a potential complication of MNB, is an abnormal collection of blood outside of a blood vessel. It occurs because the wall of a blood vessel, artery, vein, or capillary has been damaged causing blood to leak into the surrounding tissues. If a hematoma develops, it can cause localized swelling, pain, and discoloration at the injection site. Treatment options for hematoma include observation, cold compression, compression bandages, and, in severe cases, evacuation of the hematoma through aspiration or surgical drainage [15].

Infection: Infection is a potential complication of any invasive procedure, including MNB. While the risk of infection associated with MNB is generally low, it is essential to consider and address this possibility. Infection may extend to tissues by penetration of the needle through a contaminated tissue, because of the needle being contaminated before an operation. A latent viral infection may be reactivated due to the trauma of the procedure, which may be responsible for neural sheath inflammation. Antiseptic mouthwash solutions such as chlorhexidine gluconate should be taken for all regional techniques. The LA should not be injected through the infected area.

Infection causes the local pH in the infected area to decrease. The LA injected into an infected area ionizes and is less able to enter the cell membrane. This condition is what can be referred to in a clinical scenario as a “failed block” when attempting to inject anesthetics into infected tissue.

Signs and symptoms of infection following an MNB may include increased pain, swelling, redness, warmth, and the presence of pus or drainage at the injection site. Systemic symptoms such as fever or malaise may also be present. Prompt recognition and management of infection are crucial. Treatment options may include antibiotics, wound care, and drainage of any abscesses if necessary [15,103].

Mandibular nerve

It is most utilized following infiltration and is probably the most significant method in dentistry [106]. The mandibular nerve is the main division of the trigeminal nerve. The mandibular nerve is unique in that it contains both sensory and motor fibers. It provides sensory innervation of the buccal mucosa, mandibular teeth, and the skin below the mouth. The motor portion of V3 innervates all the muscles of mastication. The mandibular nerve exits the skull through the foramen ovale [107]. The MNB involves the block of inferior alveolar, auriculotemporal, buccal, incisive, mental, lingual, and mylohyoid nerves [108].

Damage to any of the above nerves can lead to numbness, loss of taste, or pain in the lip mucosa and tongue.

Complications of Mandibular Nerve Block

Hypersensitivity (allergy): Allergy, also called hypersensitive reactions, is triggered by acquired immunological mechanisms following exposure to an allergen. Upon re-exposure, individuals develop an enhanced sensitivity, leading to heightened reactions [109]. Allergic reactions caused by local anesthetic agents are recognized to involve two kinds of reactions: immunoglobulin-E-mediated is type-1 while T-cell-mediated is type-4 reaction [110]. In the meantime, late-type IV reactions primarily result from the utilization of topical anesthetics and are due to localized edema development. The prevalence of unfavorable outcomes due to local anesthetic agents is normally described as 0.1-1% [111]. The actual recognized cases of allergies among these unfavorable outcomes account for below 1%, highlighting that allergic reactions are very uncommon [112]. The allergic reactions could comprise mild symptoms, for instance, itching, erythema, and urticaria, as well as stern reactions in the shape of respiratory distress and angioedema. Furthermore, the most severe and life-threatening anaphylactic reactions comprise the indications of apnea, hypotension, and consciousness loss [109,112].

Toxicity (Overdose)

Patients in younger age groups face an elevated susceptibility to adverse drug reactions [113,114]. The majority of adverse drug events occur during the injection or for five to 10 minutes [115]. The systemic toxicity caused by local anesthetic agents can occur when elevated blood levels are observed, either through a single unintentional intravascular injection or recurrent injections [116]. Administration of local anesthetic leads to a biphasic reaction within the CNS. Generalized tonic-clonic convulsion is the typical reaction of overdose reaction to local anesthetic [117]. Initial subjective signs of toxicity primarily involve CNS and encompass dizziness, confusion, and anxiety. It could be followed by drowsiness, tinnitus, diplopia tingling, or circumoral numbness. The objective indications could comprise muscle twitching, slow speech, talkativeness, shivering, and tremors, after that explicit seizure activity. Respiratory arrest and unconsciousness could also occur [118].

The response of the cardiovascular system (CVS) to the local anesthetic agent toxicity is characterized by a biphasic pattern as well. During the initial phase, the CVS undergoes stimulation, leading to increases in BP and heart rate. With rising plasma levels of anesthetic, vasodilation takes place, which is subsequently accompanied by myocardial depression with a subsequent decrease in blood pressure. Cardiac arrest and bradycardia could occur. The local anesthetic agents’ cardio-depressant effects are not observed until an elevated level is found in the blood [119].

The toxicity of local anesthetics could be averted by meticulous injection methods, watchful observation, and knowledge regarding the maximum dose based on the patient’s body weight. It is important to acknowledge that when following the same dosing recommendation, a 4% local anesthetic agent requires half volume compared to a 2% solution [119]. Aspiration during injections decreases the intravascular injection risk while employing a slow injection approach minimizes tissue distortion as well as associated discomfort. Following injection, a doctor, assistant, or hygienist needs to stay with the patient during the initial onset of the anesthetic's effect. Timely identification of toxic reactions is significant for efficient management. Once signs and indications of toxicity are noticed, the local anesthetic drugs should be stopped [120].

Hematoma

It could occur in the pterygomandibular space due to bleeding caused by needle prick and is an uncommon problem. Patients could have pain, inflammation, and problems in the mouth opening. The treatment primarily involves symptomatic care and, in some cases, prophylactic antibiotic coverage. The condition is expected to resolve on its own [2].

Trismus

Trismus, which is a decrease in the range of the mandibular motion, can take place following a dental injection. Another potential cause of trismus is hematoma accumulation, which hinders the ability to perform excursive movements necessary for complete opening. During the trismus acute phase after dental injection, the primary treatment approaches involve the usage of analgesics and a soft diet. If needed, gradual restoration of normal function and physiotherapy can be implemented [121].

Paresthesia

Paresthesia refers to the presence of prolonged anesthesia beyond anticipated duration [113]. It can occur due to nerve trauma and one of the potential causes, among others, is the needle used during injection. During injection, individuals who encounter initially electric shock sensations could develop constant anesthesia [122]. It has been described that paresthesia is more frequently observed with 4% solutions like prilocaine and articaine when compared with solutions containing lesser concentrations [34]. Some reasons suggested for this include the fact that these solutions have a concentration higher than 2%, and a high number of articaine and prilocaine-related paresthesia instances have been documented when compared to other LA of lesser concentration. Perhaps the dentist's personal preference for the type of LA to be used in blocks is also a possible reason. In addition, commercial articaine is routinely combined with epinephrine, which increases the risk of neurotoxicity caused by articaine while epinephrine is generally not added to other common commercial local anesthetics in dentistry.

Neuralgia

It is referred to episodic, severe intermittent pain that typically occurs within neck and head nerve-specific branches. The cranial nerve V is accountable for the sensory innervation of the face, mouth, and scalp, as well as damage/disease to this nerve could cause pain or sensory loss. Trigeminal neuralgia (TN), commonly known as "tic douloureux," is considered one of the most severe and acknowledged neuralgias. It differs from facial tics, as facial tics are uncontrollable spasms in the face and can lead to rapid eye blinking or nose scrunching. They may also be called mimic spasms. Although facial tics are usually involuntary, they may be suppressed temporarily. It is not clear what causes tics. They are thought to be due to changes in the parts of the brain that control movement. They can run in families, and there is likely to be a genetic cause in many cases. Tic douloureux is characterized by distinct symptoms of sharp, intense, stabbing sensations (similar to electric shocks) with or without burning sensation that can affect the entire face. It is recognized as the most persistently painful condition experienced in the human body. The pain, triggered even by a gentle touch to a specific area of the skin, can manifest unpredictably and at any moment. The frequency of these attacks may differ depending on the severity of the condition [70].

This intense medical condition has an impact on one or multiple branches of the fifth cranial nerve. Above 85% of TN cases are of the classic type recognized as classical TN; however, the remaining cases could be separated into secondary TN. The secondary TN is believed to be triggered by multiple sclerosis or the presence of a space-occupying lesion that affects the cranial nerve V. On the other hand, classical TN is primarily caused by trigeminal nerve compression within the area of the dorsal root entry zone through blood vessels. Pain can be associated with tumors affecting the trigeminal nerve pathways. The most frequent are meningiomas and vestibular schwannomas (VS). On the other hand, classical TN is primarily caused by trigeminal nerve compression within the area of the dorsal root entry zone through the superior cerebellar artery (SCA) [70].

Facial nerve

It is the 7th cranial nerve that emerges from the skull through the stylomastoid foramen. Before this, a branch of chorda tympani arises, providing preganglionic secretomotor fibers to sublingual and submandibular glands. Also, it transports the motor efferent fibers responsible for taste from the anterior 2/3rd of the tongue, excluding vallate papillae. The facial nerve after leaving the skull divides into two major branches (cervical and temporal) before arriving at the parotid gland, in which it separates into cervical, mandibular, buccal, temporal, and zygomatic branches that finally supply the facial expression muscles [99].

Bell’s Palsy (Facial Nerve Palsy)

**Figure 2:**
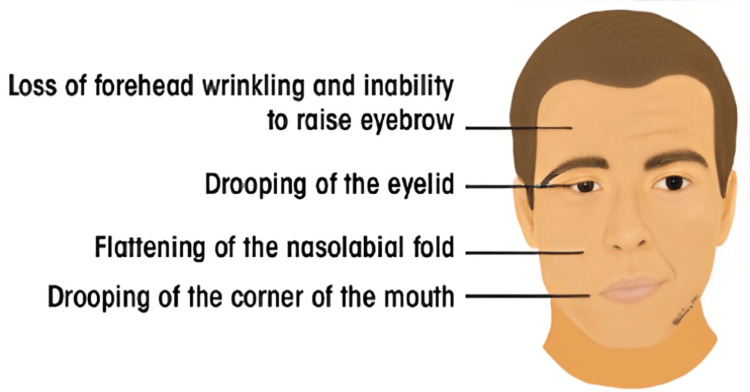
Signs of acute unilateral Bell’s palsy. Used with permission from [125].

**Figure 3:**
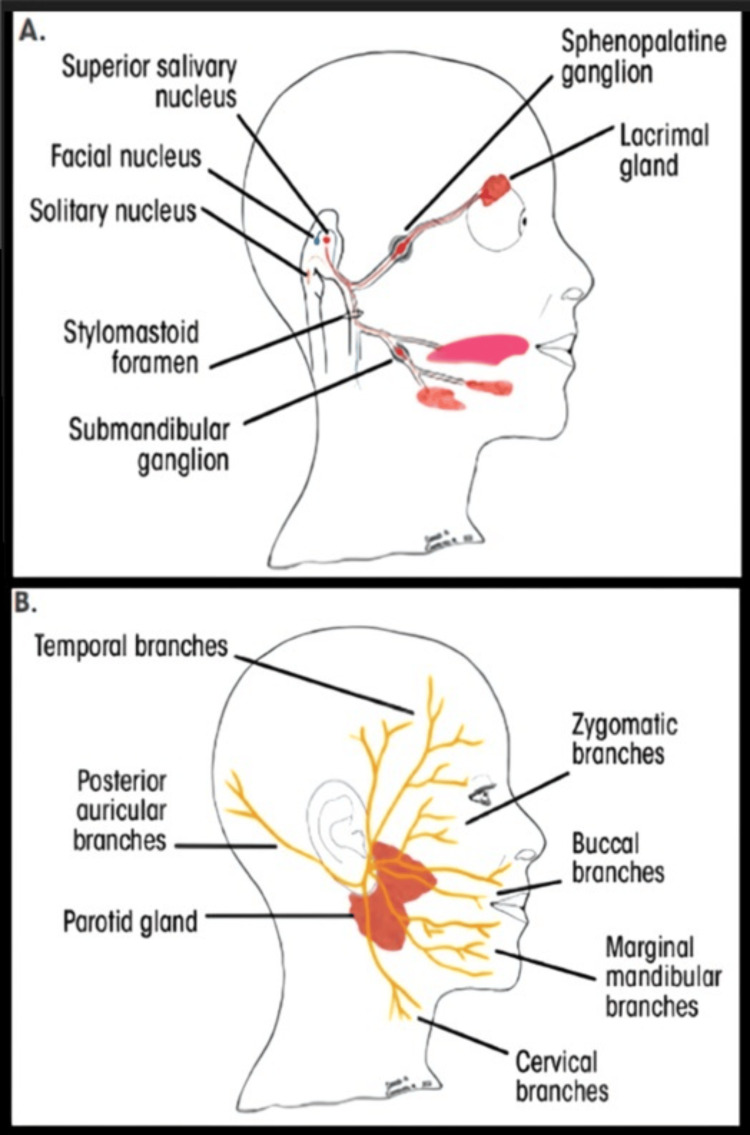
(A) Structures innervated by the facial nerve. The red (parasympathetic), blue (motor), and orange (sensory) lines demonstrate the multifunctionality of the facial nerve. (B) Superficial branches of the extracranial portion of the facial nerve. Used with permission from [125].

The most frequent neurological complication after IANB is FNP (Figures 2, 3) [96,123-125].

Patients with peripheral FNP show the following symptoms: generalized weakness of the ipsilateral side of the face; incapability to close eyelids; nasolabial fold obliteration; drooping of mouth corner; and mouth deviation toward the unaffected side.

They could also report pain within the area behind the ear and a reduced sense of taste [99]. The FNPs can have either peripheral or central origins. In the case of unilateral paralysis, the differentiation is made by observing that a lesion of the upper motor neuron leaves the forehead unaffected muscles. This is because the forehead obtains innervation by both cerebral hemispheres, caused by crossing over of the fibers within corticonuclear tracts. In contrast, peripheral nerve palsy is due to an insignificant motor neuron lesion, which results in the involvement of all facial muscles [99].

The FNP that occurs after IANB can manifest either immediately or with a delay [99].

Immediate Palsy

Generally, the immediate palsy recovers in three hours after local anesthetic agent administration. It is possibly caused by the facial nerve trunk due to nerve abnormal anatomy, for example, the passage of the nerve together with the parotid gland's deep surface. Alternatively, it could be due to an inherited abnormality, for example, the gland failing to envelop the nerve as well as its divisions, hence, enhancing its risks of direct exposure to the local anesthetic agent. Also, it has been suggested that the parotid gland capsule will avert any escape of the local anesthetic drug unintentionally deposited in the gland substance, hence, maintaining an elevated concentration of the solution in contact with passing branches of the facial nerve [126].

However, these explanations do not account for chorda tympani involvement and the related taste disturbance, the incidence of FNP after posterior alveolar nerve block, and the delayed onset of palsy several hours after the anesthesia has subsided [99].

Delayed Palsy

Delayed-onset facial palsy takes place several hours (in some cases several days) following the anesthetic agent administration. Three hypotheses have been proposed to elucidate this phenomenon [99].

**Figure 4:**
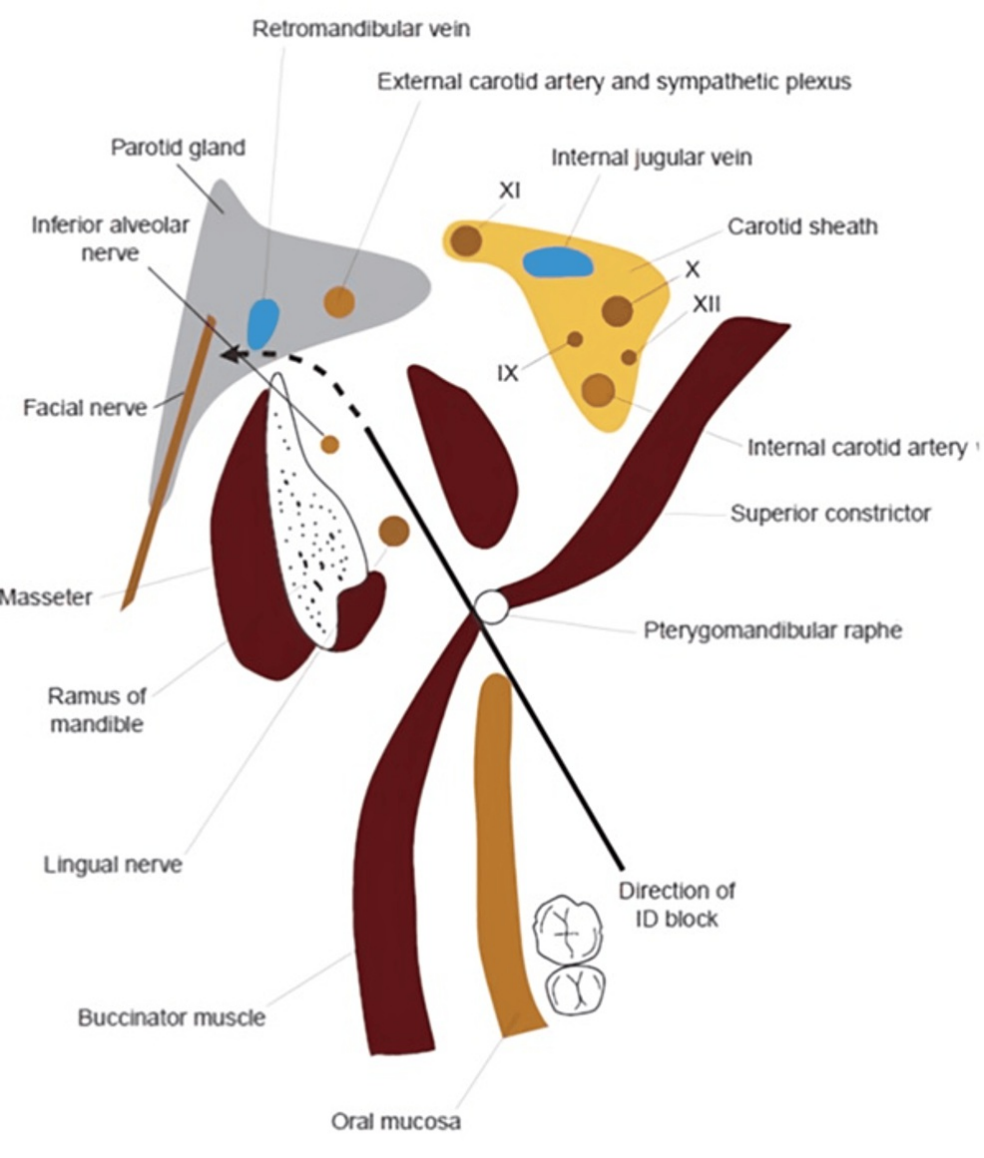
Transverse section through the retromandibular region to demonstrate the path of direction of the needle during an inferior dental (ID) nerve block. The potential direction that deposited local anesthetic solution may take to enter the substance of the parotid gland, and consequently, the facial nerve is marked with a dotted line. Used with permission from [99].

The anesthetic or its breakdown products excite the sympathetic plexus related to the external carotid artery (Figure 4).

Originating from the external carotid artery, fibers of this plexus persist in conjunction with the stylomastoid artery. In about 66% of the cases, the stylomastoid artery is a branch of the occipital artery, while in the remaining cases, it is a branch of the auricular artery, as it traverses through the parotid gland. The activation of the stylomastoid sympathetic plexus triggers a delayed reflex spasm of blood vessels supplying the facial nerve, resulting in ischemic neuritis as well as subsequent edema. The superior cervical ganglion serves as a source of these sympathetic fibers that then give rise to anterior, lateral, and medial directions. Among these branches, it is the anterior branch that extends toward common as well as external carotid arteries forming plexuses that travel together with the blood vessels [127].

The needle's mechanical action itself could cause stimulation of the sympathetic plexus related to the external carotid artery [126]. Latent viral infectivity reactivation caused by trauma of procedure could be accountable for neural sheath inflammation as well as subsequent disruption in function [124].

The proximity to the external carotid artery and associated sympathetic plexus should be noted.

Transient Amaurosis

Another, more terrifying complication, transient amaurosis (blindness), has been reported following an inferior dental block [128,129].

Complications of other nerve blocks

Other blocks such as mental or infraorbital nerve blocks along with soft tissue infiltration can lead to adverse effects but no complications have been reported [99].

## Conclusions

Neurological complications associated with LA are relatively rare but can occur. The potential risks include nerve injury, neurotoxicity, and systemic toxicity. The overall incidence of these complications is low. However, utilization of proper techniques, appropriate dosages, and meticulous monitoring can help minimize the risks. Healthcare professionals who administer LA should be well-trained and knowledgeable about the potential complications and their management.

If any neurological complication arises during or after an LA procedure, prompt recognition and appropriate management are crucial. This may include discontinuing the administration of anesthetic, providing supportive care, and, if necessary, consulting with a specialist/neurologist for further evaluation and treatment.
